# ZD1839 (‘Iressa’), a specific oral epidermal growth factor receptor-tyrosine kinase inhibitor, potentiates radiotherapy in a human colorectal cancer xenograft model

**DOI:** 10.1038/sj.bjc.6600182

**Published:** 2002-04-08

**Authors:** K J Williams, B A Telfer, I J Stratford, S R Wedge

**Affiliations:** School of Pharmacy and Pharmaceutical Sciences, University of Manchester, Manchester M13 9PL, UK; Cancer and Infection Bioscience, AstraZeneca, Alderley Park, Macclesfield, Cheshire SK10 4TG, UK

**Keywords:** ZD1839, EGFR-TKI, radiotherapy, LoVo xenograft, Iressa

## Abstract

The effect of ZD1839 (‘Iressa’), a specific inhibitor of the tyrosine kinase activity of the epidermal growth factor receptor, on the radiation response of human tumour cells (LoVo colorectal carcinoma) was evaluated *in vitro* and *in vivo*. ZD1839 (0.5 μM, incubated days 1–5) significantly increased the anti-proliferative effect of fractionated radiation treatment (2 Gy day^−1^, days 1–3) on LoVo cells grown *in vitro* (*P*=0.002). ZD1839 combined with either single or fractionated radiotherapy in mice bearing LoVo tumour xenografts, also produced a highly significant increase in tumour growth inhibition (*P*⩽0.001) when compared to treatment with either modality alone. The radio-potentiating effect of ZD1839 was more apparent when radiation was administered in a fractionated protocol. This phenomenon may be attributed to an anti proliferative effect of ZD1839 on tumour cell re-population between radiotherapy fractions. These data suggest radiotherapy with adjuvant ZD1839 could enhance treatment response. Clinical investigation of ZD1839 in combination with radiotherapy is therefore warranted.

*British Journal of Cancer* (2002) **86**, 1157–1161. DOI: 10.1038/sj/bjc/6600182 www.bjcancer.com

© 2002 Cancer Research UK

## 

The epidermal growth factor receptor (EGFR) is a transmembrane glycoprotein consisting of an external ligand binding domain, a transmembrane domain and an intracellular tyrosine kinase domain. EGFR activates downstream signalling pathways involved in cell proliferation and survival, such as the Ras/Raf/MAPK and the PI3-K/Akt pathways, in response to the binding of structurally related ligands such as EGF and transforming growth factor α (TGFα) (reviewed in Salomon *et al*, 1995). Expression or overexpression of EGFR is frequently observed in human cancers including non-small cell lung cancer and hormone-refractory prostate cancer (Salomon *et al*, 1995), and over expression has been associated with poor prognosis in several cancer types, including breast (Sainsbury *et al*, 1987), pancreas (Dong *et al*, 1998) and laryngeal (Maurizi *et al*, 1996). This observation provided the rationale for the development of inhibitors of the EGFR-mediated signalling cascade as anticancer therapeutics (Ciardiello, 2000a). ZD1839 (‘Iressa’) is an orally active, selective EGFR-TKI (epidermal growth factor receptor-tyrosine kinase inhibitor) that blocks signal transduction pathways implicated in the proliferation and survival of cancer cells, and other host-dependent processes promoting cancer growth. ZD1839 is currently in Phase III development for non-small cell lung cancer and in Phase II clinical trials in a number of other tumour types, and has demonstrated promising antitumour activity with good tolerability in Phase I dose-escalation studies (Baselga *et al*, 2000; Ferry *et al*, 2000). Recently, *in vitro* studies have revealed that ionising radiation stimulates EGFR-mediated MAPK activation (Schmidt-Ullrich *et al*, 1997). Enhanced signalling via this pathway affords a proliferative (Schmidt-Ullrich *et al*, 1997; Dent *et al*, 1999; Reardon *et al*, 1999) and survival advantage (Dent *et al*, 1999) in response to radiation treatment. The purpose of the present study was to evaluate whether ZD1839 overcomes this resistance pathway and hence influences the response of a human colorectal carcinoma cell line (LoVo) to radiation treatment *in vitro* and when grown as tumour xenografts in nude mice.

## MATERIALS AND METHODS

### Cell line details

The LoVo human colorectal carcinoma cell line was obtained from ECACC. All cell culture reagents were obtained from Gibco BRL. Cells were maintained in RPMI medium supplemented with 10% foetal calf serum and 2 mM glutamine and routinely screened for the presence of mycoplasma (Mycotect assay; Gibco BRL).

### *In vitro* studies

The procedure used for the *in vitro* evaluation of ZD1839 in combination with radiation was adapted from previously published methods (Dent *et al*, 1999). Exponentially growing LoVo cells were sub-cultured into duplicate 24-well plates (Falcon) at 5×10^−3^ cells per well and left for 24 h. Cells in both plates were exposed to ZD1839 at a range of concentrations (0.05–0.5 μM) diluted from a 10 mM stock in DMSO using pre-warmed RPMI medium. Thirty minutes after drug exposure, one plate was irradiated with 2 Gy (X-ray, delivered at a dose rate of 0.75 Gy per min) and the second sham-irradiated. The radiation procedure was performed again 24 and 48 h later (total dose 6 Gy). Prior to the final dose, freshly prepared ZD1839 was applied to the plates and the plates incubated for 72 h. The replenishment of medium and ZD1839, 48 h through the 5 day period was performed to ensure consistent drug exposure for the duration of the assay, since our preliminary experiments with ZD1839 alone were based on a 72 h incubation and previous experiments with ZD1839 *in vitro* have used drug replenishment (Ciardiello *et al*, 2000b). Cell proliferation was then analysed by MTT (3-4,5 dimethylthiazol-2, 5 diphenyl tetrazolium bromide) assay (Carmichael *et al*, 1987). MTT (1 mg ml^−1^ in PBS) was added to each experimental well to a final concentration of 0.5 mg ml^−1^. Cells were incubated for 4 h at 37°C, the MTT containing medium was removed and 1 ml DMSO added per well. Plates were shaken and the optical density (OD) read at 540 nm using a multiwell plate reader (Titertek Twinreader).

In data analysis, pooled OD_540nm_ values from replicate experiments were used to determine drug or radiation effects on proliferation. Drug-dependent growth inhibition was calculated relative to either untreated (sham-irradiated) or radiation only treated controls, in individual experiments, and the mean value from 4–5 replicate experiments calculated.

### Xenograft studies

Exponential phase LoVo cells were prepared at a 5×10^7^ per ml concentration in serum-free RPMI medium. Xenografts were established by the intra-dermal injection of a 0.1 ml volume of the prepared cell stock, 1 cm from the tail base on the midline of female nude mice (cba nu/nu) aged 8–10 weeks. Once a palpable tumour was apparent, tumour volume was measured daily, using calipers. Radiotherapy was administered at a dose rate of 2 Gy per min to unanaesthetised mice restrained in polyvinyl jigs with lead shielding and a cut away section to allow local irradiation of the tumour by the unilateral beam (Pantac X-ray set; Sheldon and Hill, 1977). Jigs were turned through 180° halfway through the radiation exposure time to provide a uniform dosing. For ethical reasons, experiments were terminated when a relative tumour volume four times that at the initiation of therapy (RTV_4_) was achieved. All procedures were carried out in accordance with the Scientific Procedures Act 1986 by approved protocols (Home Office Project License number 40–1770) and with ethical committee approval. The ethical guidelines that were followed meet the standards required by the UKCCCR guidelines (Workman *et al*, 1998). Initial experiments were performed to ascertain the response of the LoVo xenograft to single-dose radiotherapy. Tumours were irradiated at 5, 10 and 15 Gy to obtain a dose response curve for this xenograft model. The anti-tumour effect of ZD1839 in combination with single- and fractionated-dose radiotherapy was addressed in two separate protocols. Tumour-bearing mice were randomly assigned into groups to receive either ZD1839 (100 mg kg^−1^) or 0.5% polysorbate vehicle, once daily by oral administration (0.1 ml per 10 g body weight) for 14 days. This was administered alone, or combined with a single X-ray dose of 5 Gy (Protocol 1) or three multiple fractions of 2 Gy (Protocol 2) delivered at 24 h intervals. All treatments were performed on mice bearing xenografts of 220–280 mm^3^ in volume. Radiotherapy was administered 2 h after ZD1839 or vehicle dosing. Growth delays were calculated from the difference in time taken to achieve RTV_4_ between control and treated tumours.

### Statistical analyses

Mann–Whitney *U* or 2-tailed paired Student's *t*-tests were used to evaluate the significance of the results obtained. Statistical analysis was performed using SPSS for Windows (Version 8).

## RESULTS

### Antiproliferative effect of ZD1839 in combination with radiation in LoVo human colorectal cancer cells treated *in vitro*

**Figure 1:**
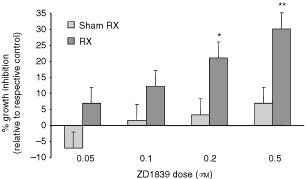
Enhanced growth-inhibitory effect of ZD1839 when combined with radiation *in vitro*. ZD1839-induced growth inhibition was examined in the LoVo cell line with (Rx) or without (sham Rx) additional radiation treatment. LoVo cells were exposed to ZD1839 for a period of 6 days, with or without 3×2 Gy radiation administered on days 1–3 of drug treatment. Cells were then treated with MTT for 4 h, lysed with DMSO and the optical density (OD) of the resulting solution determined using a multi-well plate reader. Growth inhibition normalised to the relevant control (i.e. ZD1839 treatment alone related to sham-treated control and ZD1839+radiation related to treatment with radiation alone) was calculated in each independent experiment. Data presented are means (s.e.m.) from 4–5 individual experiments. *P=0.03; **P=0.02 (2-tailed and *t*-test).

Recent studies have shown that inhibition of EGFR-linked MAPK activation can have profound effects on proliferation following radiation treatment *in vitro* (Dent *et al*, 1999; Reardon *et al*, 1999). To ascertain whether inhibition of the TK activity of EGFR using ZD1839 influences the proliferation of LoVo cells following radiation, cells were exposed to ZD1839 continually for 6 days, either alone, or in combination with three 2 Gy doses of radiation administered at 24 h intervals on days 1–3 of the experiment. In keeping with previous studies (Dent *et al*, 1999; Reardon *et al*, 1999), relative proliferation in irradiated *vs* sham-irradiated controls was evaluated by MTT assay. In the sham-irradiated controls, the doses of ZD1839 used had no significant effect on proliferation even at the highest concentration examined (0.5 μM; *P*=0.35; Figure 1

). Treatment with radiation alone (3×2 Gy) reduced cell proliferation although this did not reach statistical significance (*P*=0.14). However, the combination of ZD1839 with irradiation led to a drug dose-dependent increase in growth inhibition compared with that seen following radiation alone (Figure 1), which was statistically significant at 0.2 and 0.5 μM drug concentrations (*P*=0.03 and 0.002, respectively; 2-tailed *t*-test).

### Radiation response of LoVo cells when grown as xenografts in nude mice

**Figure 2:**
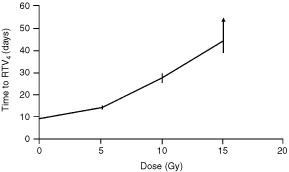
Radiation-induced growth delay in LoVo tumour xenografts. Localised radiotherapy was administered to tumours of size 220–280 mm^3^ with five allocated per treatment group. Experiments were terminated when RTV_4_ was achieved. The arrow on the 15 Gy data point indicates that the mean time to achieve RTV_4_ was actually greater than 44 days as the whole experiment was terminated 50 days after treatment, at which point RTV_4_ had been achieved in only three of the five mice treated with this dose. The data represent mean values (s.e.m.).

Our *in vitro* observations supported an evaluation of the effect of ZD1839 on the radiation response of LoVo cells when grown as xenografts in nude mice. Initial studies were undertaken to ascertain the inherent radioresponsiveness of the LoVo xenograft model. Tumours of size 220–280 mm^3^ were irradiated at 5, 10 and 15 Gy and the time to achieve RTV_4_ determined following each dose (Figure 2

). The average (s.e.m.) growth delay achieved was 5.0 (±1.2) and 18.5 (±2.2) days for the 5 and 10 Gy groups, and more than 35 days for the group treated with 15 Gy. For the latter group, the experiment was terminated 50 days after treatment, when RTV_4_ had been achieved in three of the five treated tumours.

### ZD1839 enhances the response of LoVo xenografts to radiotherapy *in vivo*

**Figure 3:**
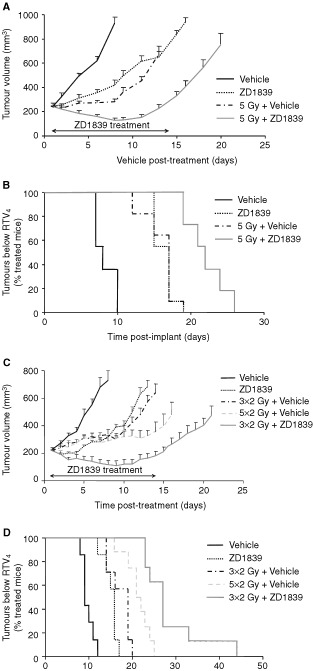
ZD1839 improves the anti-tumour effect of both single (**A** and **B**) and fractionated (**C** and **D**) dose radiotherapy in LoVo tumour xenografts. ZD1839 (100 mg kg day; po) or vehicle (0.5% polysorbate) was administered for 14 days either alone or combined with a single dose of 5 Gy (**A** and **B**) or three fractions of 2 Gy at 24 h intervals (**C** and **D**). Radiation was administered 2 h after the first dose of ZD1839 or vehicle and tumour size monitored until RTV_4_ was achieved. **A** and **C** represent the mean size (s.e.m.) of the tumours within each treatment group (*n*=7–11, see Table 1 for details), up until the point when any tumour within the treatment group reached RTV_4_. The bar indicates the period of ZD1839 dosing. **B** and **D** give a diagrammatic representation of the time taken for each individual tumour within the treatment groups to achieve RTV_4_.

To ascertain whether ZD1839 had any effect on the response of LoVo tumours to single-dose radiotherapy, a 5 Gy radiation dose was selected for use in combination with 100 mg kg^−1^ ZD1839. Previous studies had demonstrated that this dose of drug administered daily for 14 days would give approximately the same growth delay as that achieved with 5 Gy in the LoVo xenograft model (data not shown). The response of the LoVo xenografts to 5 Gy plus ZD1839 was significantly enhanced compared with that seen with either drug or radiotherapy alone (Figure 3A

,B, Table 1

**Table 1:**
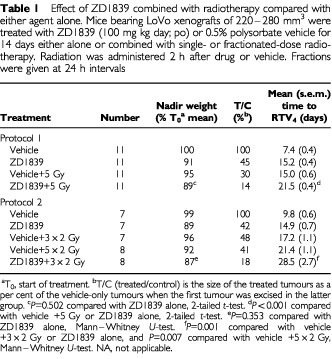
Effect of ZD1839 combined with radiotherapy compared with either agent alone. Mice bearing LoVo xenografts of 220–280 mm^3^ were treated with ZD1839 (100 mg kg day; po) or 0.5% polysorbate vehicle for 14 days either alone or combined with single- or fractionated-dose radiotherapy. Radiation was administered 2 h after drug or vehicle. Fractions were given at 24 h intervals

). From the LoVo radiation dose response curve, the time to achieve RTV_4_ for ZD1839 plus 5 Gy was equivalent to that which would be obtained for a single dose of 8 Gy, giving a radiation dose modification factor of 1.6 for the combined therapy.

The effect of 100 mg kg^−1^ ZD1839 for 14 days was then evaluated when combined with a fractionated radiation dosing protocol. Again, tumours treated with three 2 Gy fractions at 24 h intervals in combination with ZD1839 showed a significantly better response than those treated with radiation or drug alone (Figure 3C,D, Table 1). This was also the case when compared with tumours treated with 2 Gy fractions for a period of 5 days.

While the doses of radiation used were too low to elicit any normal tissue damage within the radiation field, some body weight losses were associated with ZD1839 treatment alone (Table 1). Encouragingly, no significant difference was apparent between the weight-loss observed in mice receiving only ZD1839 and those receiving the combined treatment (Table 1). In general, the nadir weight was seen 7–9 days into the 14 day treatment period. In approximately 85% of animals treated with ZD1839, with or without combined irradiation, there was a gradual recovery of weight through the remainder of the ZD1839 dosing period. The weight of the remaining animals stabilised and then increased once drug dosing had been stopped.

## DISCUSSION

The EGFR has emerged as a key target for anticancer therapeutics. This view has evolved through studies correlating EGFR expression with disease progression in multiple tumour types (Sainsbury *et al*, 1987; Salomon *et al*, 1995; Maurizi *et al*, 1996; Dong *et al*, 1998) and evidence that EGFR-inhibition can influence a diverse range of pathways, including those involved in proliferation, reduced apoptosis, angiogenesis and invasion (Woodburn, 1999). Approaches to inhibit EGFR activation include the development of anti-EGFR antibodies (Mendelsohn, 1997 (review); Yang *et al*, 1999) or small molecule inhibitors of the EGFR-tyrosine kinase (Ciardiello, 2000a), such as ZD1839.

EGFR is stimulated by a number of autocrine growth factors, including EGF and TGFα. Recently, it has been shown that ionising radiation stimulates EGFR activation and cellular proliferation (Schmidt-Ullrich *et al*, 1997; Dent *et al*, 1999; Reardon *et al*, 1999; Todd *et al*, 1999) and that anti-EGFR antibody approaches can improve tumour radiation response (Saleh *et al*, 1999; Bianco *et al*, 2000; Milas *et al*, 2000). In the present study, we evaluated the potential influence of ZD1839 on the radiation response of the human colorectal carcinoma cell line LoVo which has moderate levels of EGFR expression (33 000 receptors per cell), and is therefore likely to be representative of most epithelial tumour cell lines (Caraglia *et al*, 1994).

Initial *in vitro* studies demonstrated a clear antiproliferative effect of ZD1839 when combined with fractionated radiation treatment, using doses of ZD1839 that had no effect on cell growth when administered alone. These findings are in accordance with previously published studies, where radiation response was enhanced when combined with a dominant negative inhibitor of EGFR function (Contessa *et al*, 1999; Reardon *et al*, 1999).

A significant increase in antitumour activity was observed when ZD1839 was combined with radiotherapy *in vivo*. ZD1839 significantly enhanced the response of LoVo xenografts to both single-, and fractionated-dose radiotherapy (*P*⩽0.001, RTV_4_ values, Table 1). The effect of ZD1839 when combined with single-dose radiotherapy was equivalent to that obtained using a 60% increase in radiation dose. This dose modification would be translated into therapeutic gain if the normal tissue toxicities of radiation and ZD1839 do not overlap. It compares favourably with recent studies where radiation has been used in combination with cytotoxic agents, for example, gemcitabine, where a dose modification of 1.54 was achieved (Milas *et al*, 1999). The enhanced antitumour effect of fractionated radiotherapy when combined with ZD1839 was even more marked, with concommitant ZD1839 administration being significantly better than a 67% increase in total radiation dose.

EGFR activation in response to radiation *in vitro* has been shown to be biphasic (Dent *et al*, 1999). The secondary response appears to be dependent on radiation-induced pro-TGFα cleavage and autocrine action of TGFα*. In vitro* studies fail to demonstrate an antiproliferative effect of EGFR inhibition (using a dominant negative) following treatment with single doses of radiation (Contessa *et al*, 1999), but demonstrate clear effects in combination with fractionated radiation treatment, as seen in this and other studies (Contessa *et al*, 1999; Reardon *et al*, 1999). It is conceivable that the TGFα-mediated autocrine effects may have a greater influence *in vivo* rather than *in vitro*.

The basis of the increased activity of ZD1839 when combined with fractionated radiotherapy in tumour xenografts may result from inhibition of EGFR-mediated accelerated re-population between fractions. Indeed, EGFR signalling has been previously linked with enhanced radiation-induced proliferation in cultured cells (Schmidt-Ullrich *et al*, 1997). In addition, ZD1839 alone has been previously demonstrated to have an antiproliferative effect when administered *in vitro* that has been associated with the induction of programmed cell death (Ciardiello *et al*, 2000b).

ZD1839 was administered daily by oral dosing for a 14 day period. Weight loss observed during the administration of ZD1839 was not influenced by radiotherapy. In most ZD1839-treated mice (with or without additional radiotherapy), following a nadir mid-way through the dosing period, weight was subsequently re-gained while ZD1839 was still being administered. Previous studies examining once-daily oral administration (days 1–5 and 8–12) of ZD1839 to nude mice bred on a NCR background, have documented body weight losses of around 5% with 100 mg kg^−1^ and up to 9% with 150 mg kg^−1^ ZD1839 (Sirotnak *et al*, 2000). The slightly greater weight losses observed in the present study may reflect the greater duration of dosing and more chemo-sensitive nature of the nude mice used (cba background).

These data may have important clinical implications. ZD1839 is currently in Phase III development in non-small cell lung cancer, and has shown good tolerability and promising antitumour activity in Phase I studies in a variety of tumour types (Baselga *et al*, 2000; Ferry *et al*, 2000). The results of this study would support the use of ZD1839 in combination with radiotherapy in a clinical setting. Elevated EGFR expression has been demonstrated in lung, breast, head and neck, colorectal, oesophageal, prostate and pancreatic tumours (Salomon *et al*, 1995), in which radiotherapy (with or without surgery) is a primary treatment modality (DeVita *et al*, 1997; Yan *et al*, 2000). In addition, an inverse correlation between EGFR expression and the radiation cure rates of syngeneic murine tumours of different tissue origin has been demonstrated in preclinical studies (Akimoto *et al*, 1999). The doses of radiotherapy examined in this study were insufficient to elicit acute normal tissue toxicity in the radiation field. Provided there is no unacceptable potentiation of radiation toxicity in normal tissues, the combination of ZD1839 with radiotherapy could significantly enhance treatment response. The combination may also enable the radiotherapy dose to be reduced without diminishing curative potential, thus limiting radiation morbidity.
